# Prevalence of persistent pain after breast cancer treatment by detection mode among participants in population-based screening programs

**DOI:** 10.1186/s12885-016-2768-1

**Published:** 2016-09-15

**Authors:** Anabel Romero, Isabel Torà-Rocamora, Marisa Baré, Teresa Barata, Laia Domingo, Joana Ferrer, Núria Torà, Mercè Comas, Carmen Merenciano, Francesc Macià, Xavier Castells, Maria Sala, CAMISS Study Group

**Affiliations:** 1 Department of Epidemiology and Evaluation, IMIM-Hospital del Mar, Research Network on Health Services in Chronic Diseases (REDISSEC), Passeig Marítim 25-29, Barcelona, 08003 Spain; 2 Clinical Epidemiology and Cancer Screening, Corporació Sanitària Parc Taulí-UAB, Research Network on Health Services in Chronic Diseases (REDISSEC), Sabadell, Spain; 3 General Directorate of Health Care Programmes, Canary Islands Health Service, Las Palmas de Gran Canaria, Spain; 4 Department of Radiology, Hospital de Santa Caterina, Salt, Girona, Spain; 5 Agency for Health Quality and Assessment of Catalonia (AQuAS), Research Network on Health Services in Chronic Diseases (REDISSEC), Barcelona, Spain

**Keywords:** Breast cancer, Complications, Persistent pain, Screening, Comorbidities, Interval cancer

## Abstract

**Background:**

To date, the study of the risks and benefits of breast cancer screening has not included the onset of persistent pain after breast cancer treatment within the context of population-based screening programs. Our purpose was to investigate the prevalence of persistent pain and associated factors in women diagnosed with breast cancer (screening or interval) in the context of a population-based breast cancer screening program in Spain.

**Methods:**

A total of 1,057 women participating in a population-based breast cancer screening program were diagnosed with breast cancer between 2000 and 2008. The women were treated surgically and followed-up to 2013. The risk of developing persistent pain was estimated through multivariate logistic regression analysis.

**Results:**

Breast cancer was detected during routine screening in 732 women (69.3 %) and emerged as an interval cancer between two screening rounds in 325 (30.7 %). Persistent pain was present in 118 women (11.3 %). Women diagnosed through routine screening reported a higher prevalence of persistent pain (12.9 %) than those with interval cancers (7.8 %)(*P <* 0.05). Multivariate logistic regression analysis identified two other variables associated with persistent pain: having a Charlson index > =2 (Odds Ratio [OR]: 4.5 95 % Confidence Interval [CI]: 2.1-9.5) versus no comorbidities, and having undergone an axillary lymph node dissection (OR: 2.0 95 % CI: 1.0-4.0) versus sentinel lymph node biopsy.

**Conclusions:**

The prevalence of persistent pain was relatively low. The detection mode was not related to the onset of persistent pain. The factors associated with persistent pain were a Charlson index > =2 and the performance of axillary lymph node dissection. Women treated for breast cancer are at risk for developing persistent pain regardless of the detection mode, especially those with comorbidities and those who have undergone axillary lymph node dissection.

## Background

Breast cancer is the most frequent malignancy among Spanish women, with an estimated 25,215 new cases in 2012 [1]. Screening programs and improved treatments have reduced mortality from this disease [2]. Early detection allows more effective treatment, but the treatment itself is not free of complications in the short term or sequels in the long term. Women with interval cancer (cancers detected clinically after a negative screening round and before the following screening invitation) may not benefit from early detection because of a delay in diagnosis and less favourable biological tumor characteristics [3].

To date, complications have not been evaluated by according to the detection method. Among complications, persistent pain, defined by some authors as pain in the breast area, axilla, shoulder or arm for more than 3 months after breast cancer surgery, has been reported as one of the most frequent long-term complications [4]. Among patients treated surgically for breast cancer, the prevalence of persistent pain may range from 10 % to 50 % or more, depending on the characteristics of the population studied, as well as on the definition of persistent pain [4–16]. Several factors have been associated with the risk of developing persistent pain: young age [5–7, 10, 12, 14, 16, 17] adjuvant therapies (chemotherapy or radiotherapy) [5, 11], axillary lymph node dissection (ALND) [5–7, 10, 12], and the presence of comorbidities [5, 16], among others, but the evidence remains inconclusive.

Given that a substantial number of women are diagnosed with breast cancer and that most are treated surgically, a large number of women can be expected to experience persistent pain, which may negatively affect their quality of life and psychosocial well-being. Therefore, from the perspective of public health, persistent pain in women treated for breast cancer is an important health problem. To date, study of the risks and benefits of breast cancer screening has not included the onset of complications and sequels after breast cancer treatment within the context of population-based screening programs. Women with screen-detected breast cancer benefit from early detection and have better survival [18, 19]. However, no studies have evaluated whether these women also benefit from fewer complications and have less persistent pain in the long term. The risk-benefit analysis of breast cancer screening should include information on complications in women with screen-detected breast cancer.

We aimed to investigate the prevalence of persistent pain and associated factors in women diagnosed with breast cancer (screening or interval) in the context of a population-based breast cancer screening program in Spain.

## Methods

### Study population

Our study population was drawn from the CAMISS cohort, which includes 1,086 women with breast cancer from a population-based screening program. These women were diagnosed with breast cancer between 2000 and 2008 and were followed-up to December 2013. Breast cancer was detected in routine screening or emerged as an interval cancer. The definition of interval cancer used was that proposed in the European guidelines as “primary breast cancer arising after a negative screening episode, with or without further assessment, and before the next screening invitation, or within 24 months for women who reached the upper age limit” [20].

All women were resident in Spain, were aged 50 to 69 years at diagnosis, and were from 2 Spanish regions: the Canary Islands and Catalonia. In Spain, all women aged 50 to 69 years are actively invited to participate in the population-based screening program by a written letter every 2 years, following the European guidelines for Quality Assurance in Mammographic Screening Recommendations. This nationwide program achieves the required standards [21].

### Study variables

Interval cancers were identified by merging data from the registers of screening programs with population-based cancer registries, the regional Minimum Data Set and hospital-based cancer registries. Further details on the identification of interval cancers are reported elsewhere [3]. For the purpose of this study, only two categories were considered in the final analysis: interval or screening cancers.

Women’s age at diagnosis was obtained from the date of birth and date of the screening mammogram. The presence of other comorbidities was identified at clinical records review. All the comorbidities recorded in the clinical records at the date of breast cancer diagnosis were considered, although we only included the comorbidities needed to calculate the Charlson index [22]. Tumor-related characteristics (histological type and grade, focality, size, lymph node involvement, estrogen receptor [ER], progesterone receptor [PR], human epidermal growth factor receptor 2 [HER2] status) were obtained from the cancer registries, hospital-based registers, and from clinical records. Based on expression of ER, PR and HER2, tumors were classified into four phenotypes: 1) luminal A: ER+/HER2- OR PR+/HER2-; 2) luminal B: ER+/HER2+ or PR+/HER2+; 3) HER2: ER-/PR-/HER2+; and 4) triple-negative: ER-,PR-, HER2- [23].

Information on the treatments received was obtained from the clinical records. Two types of surgery were considered: radical or conservative. Women with radical surgery included all those who underwent mastectomies, whether radical or simple. In both cases, the breast is completely removed. In conservative surgery, only the tumor and some of the healthy surrounding tissue are excised, and the breast is fully preserved.

In addition to breast surgery, some women could undergo an ALND, also a surgical procedure that incises the axilla to identify, examine and remove lymph nodes. Sentinel lymph node biopsy (SLNB) has been proposed as an alternative to ALND, because it produces less morbidity [24]. SLNB was a relatively new technique during the study period and was introduced at different times in each hospital. It was introduced in the first hospital in 2000 and in the last hospital in 2004.

Chemotherapy, radiotherapy, hormone therapy or specific Her2 treatment may have been used as an adjuvant therapy before and/or after surgery.

### Outcome variables

The onset of breast cancer treatment complications was reviewed throughout the patient’s clinical course, starting from the date of surgery until the end of follow up. The reviewers recorded the onset of the following complications: persistent pain, lymphedema, anxiety, fatigue, disability, osteoporosis, agranulocytosis, lymphopenia, seroma, weight gain, paraesthesia, infection, necrosis, cardiomyopathy, cognitive dysfunction, pneumonitis, mycosis, hypothyroidism, renal toxicity, ototoxicity, pulmonary fibrosis and other complications not specified.

The definition of persistent pain was the following: a woman was considered to have persistent pain if she felt pain in the area of the operated breast, axilla, shoulder or arm in some of the follow-up visits at least 3 months after surgery [25]. The pain could be neuropathic or not. According to this definition, in the clinical record review, a woman was considered to have pain if, in some of the follow-up visits (at least one visit 3 months after surgery), the physician assessing her reported the presence of pain in some of the previously described areas (operated breast area, axilla, shoulder or arm). The clinical record review was performed by trained staff (nurses) at each of the participating centers.

### Statistical analysis

We performed a descriptive analysis of the study variables. The prevalence of overall complications were estimated, as well as the prevalence of persistent pain and its 95 % confidence intervals (CI). The prevalence of overall complications was computed as the number of women with at least one complication among the total number of women with information on the complications variable.

Comparisons were made between women with screening-detected cancers and those with interval cancers. Statistical significance was estimated using the chi-square or Fisher exact tests, since all the study variables were considered as categorical. The prevalence of persistent pain were described by detection mode, age at diagnosis, Charlson index, tumor characteristics and breast cancer treatments. The crude and adjusted risk of developing persistent pain was estimated through a multivariate logistic regression analysis. The multivariate logistic regression model included the following variables: detection method, age, Charlson index, histological type, phenotype, axillary treatment, neoadjuvant treatment and chemotherapy after surgery. Statistical significance was considered if the *P*-value was <0.05. Statistical analyses were performed using the SPSS statistical package (version 12.0).

## Results

Of the 1,086 women included in the CAMISS cohort, most were treated surgically (*n* = 1,057, 97.3 %) and were included in the analysis. Breast cancer was detected during routine screening mammograms in 732 women (69.3 %) and emerged as interval cancer in 325 (30.7 %). The characteristics of the women and detected tumors by detection mode, as well as the treatments provided, are shown in Table 1. Women with interval cancers had larger tumors, with a higher percentage of positive lymph node involvement (44.4 % vs. 25.8 %; *P <* 0.001). The percentage of ductal in situ tumors was higher among women with screen-detected tumors (11.1 % vs. 3.8 %; *P <* 0.001). Histological grade III was more frequent among women with interval cancers (42.3 % vs. 24.2 %; *P <* 0.001), as well as among those with triple negative phenotype (16.1 % vs. 8.1 %; *P <* 0.001). Women with interval cancer were younger than those with screen-detected cancers (*P <* 0.001). No differences in the Charlson index were observed according to the detection method. Most women underwent conservative surgery (*n* = 829, 78.6 %). According to the detection method, radical surgery was more common among women with interval cancers (33.0 %) than in those with screen-detected cancers (16.3 %) (*P <* 0.001). ALND was more common in women with interval cancers than in those with screen-detected tumors (78.4 % vs. 70.6 % *P <* 0.05). Neoadjuvant therapy was more frequent in women in the interval cancer group than in the screening group (24.0 % vs. 4.2 % *P* < 0.001).

**Table 1:** Tumor and women’s characteristics, treatments performed and frequencies of complications, according to detection method

	Screen-detected cancers		Interval cancers		*P*-value	Total	
	N	%	N	%		N	%
Total women	732	69.3	325	30.7		1057	100.0
Tumor size (*n =* 936)					<0.001		
< = 10 mm	198	31.9	40	12.7		238	25.4
11 to 20 mm	273	44.0	117	37.0		390	41.7
21 to 50 mm	132	21.3	133	42.1		265	28.3
> 50 mm	17	2.7	26	8.2		43	4.6
Lymph node involvement (*n =* 1054)					<0.001		
Negative	542	74.2	180	55.6		722	68.5
Positive	188	25.8	144	44.4		332	31.5
Number of positive nodes (*n =* 870)					<0.05		
= <3	515	92.5	268	85.6		783	90.0
4–20	38	6.8	41	13.1		79	9.1
> 20	4	0.7	4	1.3		8	0.9
Histological type (*n =* 1050)					<0.001		
Ductal Infiltrante	531	72.7	242	75.6		773	73.6
Ductal In situ	81	11.1	12	3.8		93	8.9
Lobular	63	8.6	36	11.3		99	9.4
Otros	55	7.5	30	9.4		85	8.1
Focality (*n =* 894)					0.643		
Unifocal	487	83.5	256	82.3		743	83.1
Multifocal and/or multicentric	96	16.5	55	17.7		151	16.9
Histological grade (*n =* 914)					<0.001		
I	181	28.8	48	16.8		229	25.1
II	249	39.6	104	36.4		353	38.6
III	152	24.2	121	42.3		273	29.9
Not applicable	46	7.3	13	4.5		59	6.5
Tumor phenotype (*n =* 769)					<0.001		
Luminal A	275	56.9	132	46.2		407	52.9
Luminal B	131	27.1	79	27.6		210	27.3
HER2	38	7.9	29	10.1		67	8.7
Triple negative	39	8.1	46	16.1		85	11.1
Age (*n =* 1057)					<0.001		
50–54	193	26.4	117	36.0		310	29.3
55–59	183	25.0	100	30.8		283	26.8
60–64	211	28.8	67	20.6		278	26.3
65–70	145	19.8	41	12.6		186	17.6
Charlson index (*n =* 1057)					0.380		
0	535	73.1	241	74.2		776	73.4
1	129	17.6	53	16.3		182	17.2
> =2	68	9.3	31	9.5		99	9.4
Surgery (*n =* 1055)					<0.001		
Radical	119	16.3	107	33.0		226	21.4
Conservative	612	83.7	217	67.0		829	78.6
Axillary treatment: SLNB and ALND^c^ (*n =* 994)					<0.05		
Only SLNB	201	29.4	67	21.7		268	27.0
ALND	483	70.6	243	78.4		726	73.0
Neoadjuvant therapy (*n =* 1057)					<0.001		
No	701	95.8	247	76.0		948	89.7
Yes	31	4.2	78	24.0		109	10.3
Adjuvant therapy (*n* = 1051)					0.387		
No	16	2.2	10	3.1		26	2.5
Yes	712	97.8	313	96.9		1025	97.5
Any complication^a^ (*n =* 1057)	225	30.7	88	27.1	0.229	313	29.6
Persistent pain^b^ (*n =* 1045)	93	12.9	25	7.8	<0.05	118	11.3

^a^Number of women with at least one complication

^b^Number of women with persistent pain

^c^SLNB: sentinel lymph node biopsy. In this category only women with SLNB performed were included. ﻿ALND: axillary lymph node dissection. This category includes women with only ALND performed and women with SLNB and ALND

A total of 313 women (29.6 % 95 % CI: 27.4 –33.0) experienced at least one complication during follow-up. Among all the complications reported, the most frequent was persistent pain, which was present in 118 women (11.3 % 95 % CI: 9.4 –13.2). Women who were diagnosed in routine screening reported a higher prevalence of persistent pain (12.9 % 95 % CI: 10.4 –15.3) than those with tumors that emerged as interval cancers between two screening rounds (7.8 % 95 % CI: 4.8 –10.7) (*P* < 0.05) (Table 1).

Table 2 shows the prevalence of persistent pain according to women’s characteristics, the detection method and tumor characteristics. Women aged 65–70 years had a lower prevalence of persistent pain (8.7 %) than those from other age groups (50–54 years: 11.8 %, 55–59 years: 13.2 %, 60–64 years: 10.5 %). However, these differences were not statistically significant (*P* = 0.483). A gradient was observed in the prevalence of persistent pain, according to the Charlson index. Women without other comorbidities (Charlson index = 0) had the lowest prevalence of persistent pain (8.5 %), whereas those with a Charlson index > =2 had the highest prevalence of persistent pain (21.9 %) (*P* < 0.001). According to the detection method, women with screen-detected tumors had a higher prevalence of persistent pain than women with interval tumors (12.9 % vs. 7.8 % *P* < 0.05). When tumor characteristics were analyzed, the prevalence of persistent pain was higher among women with lobular histological type tumors (14.3 %) (*P* < 0.05). No statistically significant differences in the prevalence of persistent pain were observed by tumor size, lymph node involvement, focality, histology or phenotype.

**Table 2:** Prevalence of persistent pain according to detection method, tumor and women’s characteristics

	Persistent pain^a^	
	N (%)	*P*-value
Women’s characteristics		
Age (*n =* 1045)		0.483
50–54 (*n =* 305)	36 (11.8)	
55–59 (*n =* 281)	37 (13.2)	
60–64 (*n =* 275)	29 (10.5)	
65–70 (*n =* 184)	16 (8.7)	
Charlson index (*n =* 1045)		<0.001
0 (*n =* 769)	65 (8.5)	
1 (*n =* 180)	32 (17.8)	
> =2 (*n =* 96)	21 (21.9)	
Detection method (*n =* 1045)		
Screen-detected tumors (*n =* 723)	93 (12.9)	<0.05
Interval tumors (*n =* 322)	25 (7.8)	
Tumor characteristics		
Tumor size (*n =* 932)		0.907
< =10 mm (*n =* 237)	14 (5.9)	
11 to 20 mm (*n =* 387)	28 (7.2)	
21 to 50 mm (*n =* 265)	19 (7.2)	
> 50 mm (*n =* 43)	2 (4.7)	
Lymph node involvement (*n =* 1042)		0.564
Negative (*n =* 713)	78 (10.9)	
Positive (*n =* 329)	40 (12.2)	
Number of positive nodes (*n =* 866)		0.442
= <3 (*n =* 781)	54 (6.9)	
4–20 (*n =* 77)	7 (9.1)	
> 20 (*n =* 8)	1 (12.5)	
Histological type (*n =* 1038)		<0.05
Ductal (*n =* 763)	94 (12.3)	
In situ (*n =* 93)	2 (2.2)	
Lobular (*n =* 98)	14 (14.3)	
Othter (*n =* 84)	7 (8.3)	
Focality (*n =* 891)		0.461
Unifocal (*n =* 741)	56 (7.6)	
Multifocal and/or multicentric (*n =* 150)	14 (9.3)	
Histological grade (*n =* 905)		0.101
I (*n =* 228)	19 (8.3)	
II (*n =* 348)	46 (13.2)	
III (*n =* 270)	25 (9.3)	
Not applicable (*n =* 59)	3 (5.1)	
Tumor phenotype (*n =* 763)		0.291
Luminal A (*n =* 404)	41 (10.1)	
Luminal B (*n =* 207)	12 (5.8)	
HER2 (*n =* 67)	5 (7.5)	
Triple negative (*n =* 85)	9 (10.6)	

^a^Percentages were computed as follows: number of women with persistent pain divided by the total number of women for each category

Table 3 shows the prevalence of persistent pain according to the treatments provided. The prevalence of persistent pain was higher among women who underwent ALND than in those who underwent SLNB only (13.7 % vs. 6.7 % *P* < 0.001). The prevalence was also higher in women who received chemotherapy after surgery than those who did not (14.5 % vs. 8.4 % *P* < 0.01). No statistically significant differences in persistent pain prevalence were observed according to the type of surgery, neoadjuvant therapy, radiotherapy, hormone therapy or Her2 treatment after surgery.

**Table 3:** Prevalence of persistent pain, according to the treatments performed

	Persistent pain^a^	
	N (%)	*P*-value
Surgery (*n =* 1043)		
Radical (*n =* 224)	27 (12.1)	0.693
Conservative (*n =* 819)	91 (11.1)	
Axillary treatment^b^ (*n =* 983)		<0.01
Only SLNB (*n =* 268)	18 (6.7)	
ALND (*n =* 715)	98 (13.7)	
Neoadjuvant therapy (*n =* 1045)		0.090
No (*n =* 936)	111 (11.9)	
Yes (*n =* 109)	7 (6.4)	
Adjuvant therapy post-surgery (*n =* 1039)		1
No (*n =* 26)	3 (11.5)	
Yes (*n =* 1013)	115 (11.4)	
Chemotherapy (*n =* 1018)		<0.01
No (*n =* 513)	43 (8.4)	
Yes (*n =* 505)	73 (14.5)	
Radiotherapy (*n =* 998)		0.457
No (*n =* 155)	15 (9.7)	
Yes (*n =* 843)	99 (11.7)	
Hormone therapy (*n =* 1019)		0.328
No (*n =* 219)	29 (13.2)	
Yes (*n =* 800)	87 (10.9)	
Her2 treatment (*n =* 1028)		0.790
No (*n =* 979)	112 (11.4)	
Yes (*n =* 49)	5 (10.2)	

^a^Percentages are computed as follows: number of women with persistent pain divided by the total number of women for each category

^b^SLNB: sentinel lymph node biopsy. In this category only women with SLNB performed were included. ALND: axillary lymph node dissection. This category includes women with only ALND performed and women with SLNB and ALND

The factors associated with persistent pain were described using univariate and multivariate logistic regression models (Table 4). In the univariate analysis, five factors were associated with persistent pain (detection method, Charlson index, histological type, axillary treatment and chemotherapy after surgery). In the multivariate model, only the Charlson index remained statistically significant, with the highest odds ratio (OR). Women with a Charlson index > =2 had an OR of 4.5 (95 % CI: 2.1 –9.5) with respect to women without comorbidities (Charlson index = 0). Persistent pain was also more frequent among patients with ALND than in those with SLNB only, although this result was at the limit of statistical significance (OR: 2.0 95 % CI: 1.0 – 4.0).

**Table 4:** Factors associated with persistent pain: univariate and multivariate logistic regression models

	Univariate				Multivariate^a^			
		95 % CI		*P*-value		95 % CI		*P*-value
	OR	Lower	Upper		OR	Lower	Upper	
Detection method (*n =* 1045)								
Screening	Ref.				Ref.			
Interval	0.6	0.4	0.9	0.017	0.8	0.4	1.4	0.408
Age (*n =* 1045)								
50–54	Ref.				Ref.			
55–59	1.1	0.7	1.9	0.618	1.0	0.5	2.0	0.967
60–64	0.9	0.5	1.5	0.632	0.7	0.3	1.6	0.424
65–70	0.7	0.4	1.3	0.282	0.7	0.3	1.7	0.432
Charlson index (*n =* 1045)								
0	Ref.				Ref.			
1	2.3	1.5	3.7	<0.001	2.8	1.4	5.4	<0.01
> =2	3.0	1.8	5.2	<0.001	**4.5**	**2.1**	**9.5**	<0.001
Histological type (*n =* 1038)								
In situ	Ref.				Ref.			
Ductal	6.4	1.5	26.4	<0.05	0.5	0.1	4.2	0.534
Lobular	7.6	1.7	34.4	<0.01	1.1	0.5	2.7	0.784
Other	4.1	0.8	20.5	0.082	0.6	0.2	2.1	0.422
Phenotype (*n =* 763)								
Luminal A	Ref.				Ref.			
Luminal B	0.5	0.3	1.1	0.074	0.6	0.3	1.3	0.219
Her2	0.7	0.3	1.9	0.495	0.8	0.3	2.3	0.713
Triple negative	1.0	0.5	2.2	0.903	1.1	0.5	2.6	0.800
Axillary treatment^b^ (*n =* 983)								
Only SLNB	Ref.				Ref.			
ALND	2.2	1.3	3.7	<0.05	**2.0**	**1.0**	**4.0**	<0.05
Neoadjuvant treatment (*n =* 1045)								
No	Ref.				Ref.			
Yes	0.5	0.2	1.1	0.095	0.4	0.1	1.3	0.132
Chemotherapy after surgery (*n =* 1018)								
No	Ref.				Ref.			
Yes	1.8	1.2	2.8	<0.01	1.4	0.7	2.5	0.334

^a^ *N =* 704 cases were included in the multivariate analysis. The multivariate logistic regression model included the following variables: detection method, age, Charlson index, histological type, phenotype, axillary treatment, neoadjuvant treatment and chemotherapy after surgery

^b^SLNB: sentinel lymph node biopsy. In this category only women with SLNB performed were inluded. ALND: axillary lymph node dissection. This category includes women with only ALND performed and women with SLNB and ALND

## Discussion

The prevalence of persistent pain was higher in women diagnosed within the screening program than in those with interval cancers. Our first hypothesis was that, given the benefits of screening (early diagnosis, with less aggressive tumors), the treatment of screen-detected cancer would be less intensive and, therefore, have fewer complications. Surprisingly, we observed that women diagnosed within the screening program did not benefit from fewer complications; on the contrary, they initially seemed to have more persistent pain. However, in the multivariate analysis only two factors showed a clear association with the onset of persistent pain: the presence of other comorbidities and the performance of an ALND.

The prevalence of persistent pain in our study was 11.3 %. This prevalence is relatively low compared with that in other studies, which include all diagnostic methods and women of different ages, with values ranging from 10 % to 50 % or more [4, 5, 7–12, 14, 16]. In our study, information on the presence of persistent pain was extracted through the medical records review. Women were considered to have persistent pain if they had pain (either in the arm, breast area or shoulder) 3 months after surgery, but this information had to have been registered by the physician during the clinical course. If women had been asked the question “Do you have any pain?” the prevalence could possibly have been higher than 11.3 %. Our results might be interpreted as follows: 11.3 % of women treated for breast cancer had clinically relevant pain, since pain was recorded in the medical record. In addition, some cultural differences may exist in pain perception. In a European survey of chronic pain, Spanish participants reported the lowest prevalence of chronic pain (12 %) [26]. Another possible reason for the low prevalence of persistent pain is that our patients came from screening and, therefore, had less aggressive tumors and less use of ALND than women with breast cancer of all ages, including those with a symptomatic diagnosis.

Among the risk factors described in the literature, young age is usually reported to be a risk factor for developing persistent pain [5–7, 10, 12, 14, 17]. In our study population, women aged 65–70 years had the lowest prevalence of persistent pain, but the differences were not statistically significant. Since our study population only comprised women participating in population-based breast cancer screening programs, we were unable to investigate persistent pain among women of younger ages (50 years or younger) or older ages (more than 70 years).

The presence of other comorbidities has been proposed as an associated factor in the development of persistent pain [5, 16]. In our study, we investigated the presence of other comorbidities by using the Charlson index. Women with a Charlson index equal to 2 or higher had a much higher risk of developing persistent pain. It is well established that previous painful conditions predispose patients to persistent pain after surgery [5, 7, 16]. In addition, women with several comorbidities may have more depression, anxiety and other psychosocial factors that may also be risk factors for persistent pain [5, 27].

The use of ALND has been described in the literature as a risk factor for developing persistent pain [6, 7, 10, 12, 14] and in our study remained a risk factor at the limit of statistical significance in the multivariate analysis. These studies report that women with an ALND had OR values between 2.0 and up to 7.7 and, in our study, women with an ALND had an OR of 2.0. This variability may be partly due to differences in the reference population: some studies take women without ALND as the reference, whereas others take those with SLNB as the reference category.

Some studies have found that chemotherapy is related to persistent pain [5, 11]. In our study, the univariate analysis showed that women who received chemotherapy after surgery had a higher risk of persistent pain than those who did not but this result was not statistically significant in the multivariate analysis. Therefore, chemotherapy may be associated with the onset of persistent pain, but other factors are more strongly associated with to pain.

Our study has some limitations. Pain was not evaluated with validated instruments such the Visual Analogue Scale, Numerical Rating Scale or specific pain questionnaires, and could be underestimated. Most published evidence is based on pain measurement with validated instruments and therefore our results are not directly comparable to those of studies using these instruments. However, the value of reporting pain recorded in the medical record is a valid measure for clinically relevant pain, which could be comparable to the prevalence of moderate/severe pain reported in other studies. Moreover, our study did not collect women’s height and weight, the two variables used to calculate the body mass index, a variable that has been proposed as a risk factor for developing persistent pain [14, 16]. Equally, variables related to psychosocial factors were not collected, which are well-established risk factors for persistent pain [5, 7, 9, 14]. Finally, another limitation of our study is the lack of information on previous chronic pain conditions (such as low back pain, osteoarthritis, arthritis or fibromyalgia) [5, 16] or breast pain immediately before surgery [11, 14, 16]; all of these factors have been found to be related to the onset of persistent pain. Equally, no information was gathered on the presence of acute postoperative pain, another factor that it has been proposed as a risk factor for persistent pain [7]. However, the absence of these variables might not change our results on the effect of the detection method on the development of persistent pain.

This study has some important strengths. It is the first to evaluate the onset of complications and, specifically, persistent pain among women diagnosed with breast cancer participating in population-based screening programs in Spain. Our cohort study has a long follow-up period, with a median of 8.7 (Interquartile Range: 7.2 –10.6) years.

## Conclusions

Our study shows that the detection mode (screening or interval) has no direct effect on the prevalence of complications and specifically on the onset of persistent pain. Although women with screen-detected cancer had a higher prevalence of persistent pain, the multivariate analysis showed that other factors were also associated with its onset, namely, Charlson index > =2 and the performance of an ALND.

In conclusion, the prevalence of persistent pain in our population of screened women was relatively low, and women with screen-detected cancers did not benefit from less persistent pain than women with interval cancer.

## Abbreviations

ALND: Axillary lymph node dissection
CI: Confidence intervals
ER: Estrogen receptor
HER2: Human epidermal growth factor receptor 2
OR: Odds ratio
PR: Progesterone receptor
SLNB: Sentinel lymph node biopsy

## References

[1] Fact Sheets by Population [Internet]. [cited 2016 Jul 1]. Available from: http://globocan.iarc.fr/Pages/fact_sheets_population.aspx

[2] Irvin VL, Kaplan RM (2014). Screening mammography & breast cancer mortality: meta-analysis of quasi-experimental studies. PLoS One.

[3] Domingo L, Salas D, Zubizarreta R, Baré M, Sarriugarte G, Barata T (2014). Tumor phenotype and breast density in distinct categories of interval cancer: results of population-based mammography screening in Spain. Breast Cancer Res BCR.

[4] Andersen KG, Kehlet H (2011). Persistent pain after breast cancer treatment: a critical review of risk factors and strategies for prevention. J Pain Off J Am Pain Soc.

[5] Schou Bredal I, Smeby NA, Ottesen S, Warncke T, Schlichting E (2014). Chronic pain in breast cancer survivors: comparison of psychosocial, surgical, and medical characteristics between survivors with and without pain. J Pain Symptom Manage.

[6] De Oliveira GS, Chang R, Khan SA, Hansen NM, Khan JH, McCarthy RJ (2014). Factors associated with the development of chronic pain after surgery for breast cancer: a prospective cohort from a tertiary center in the United States. Breast J.

[7] Bruce J, Thornton AJ, Powell R, Johnston M, Wells M, Heys SD (2014). Psychological, surgical, and sociodemographic predictors of pain outcomes after breast cancer surgery: a population-based cohort study. Pain.

[8] Bell RJ, Robinson PJ, Nazeem F, Panjari M, Fradkin P, Schwarz M (2014). Persistent breast pain 5 years after treatment of invasive breast cancer is largely unexplained by factors associated with treatment. J Cancer Surviv Res Pract.

[9] Belfer I, Schreiber KL, Shaffer JR, Shnol H, Blaney K, Morando A (2013). Persistent postmastectomy pain in breast cancer survivors: analysis of clinical, demographic, and psychosocial factors. J Pain Off J Am Pain Soc.

[10] Mejdahl MK, Andersen KG, Gärtner R, Kroman N, Kehlet H (2013). Persistent pain and sensory disturbances after treatment for breast cancer: six year nationwide follow-up study. BMJ.

[11] Sheridan D, Foo I, O’Shea H, Gillanders D, Williams L, Fallon M (2012). Long-term follow-up of pain and emotional characteristics of women after surgery for breast cancer. J Pain Symptom Manage.

[12] Alves Nogueira Fabro E, Bergmann A, do Amaral E, Silva B, Padula Ribeiro AC, de Souza Abrahão K, da Costa Leite Ferreira MG (2012). Post-mastectomy pain syndrome: incidence and risks. Breast Edinb Scotl.

[13] Ewertz M, Jensen AB (2011). Late effects of breast cancer treatment and potentials for rehabilitation. Acta Oncol Stockh Swed.

[14] Miaskowski C, Cooper B, Paul SM, West C, Langford D, Levine JD (2012). Identification of patient subgroups and risk factors for persistent breast pain following breast cancer surgery. J Pain Off J Am Pain Soc.

[15] Li YYO, Kong SK (2011). Persistent pain after breast cancer surgery in a Chinese population. Clin J Pain.

[16] Sipilä R, Estlander A-M, Tasmuth T, Kataja M, Kalso E (2012). Development of a screening instrument for risk factors of persistent pain after breast cancer surgery. Br J Cancer.

[17] Gärtner R, Jensen M-B, Nielsen J, Ewertz M, Kroman N, Kehlet H (2009). Prevalence of and factors associated with persistent pain following breast cancer surgery. JAMA.

[18] Redondo M, Funez R, Medina-Cano F, Rodrigo I, Acebal M, Tellez T (2012). Detection methods predict differences in biology and survival in breast cancer patients. BMC Cancer.

[19] Mook S, Veer LJ V’t, Rutgers EJ, Ravdin PM, van de Velde AO, van Leeuwen FE (2011). Independent prognostic value of screen detection in invasive breast cancer. J Natl Cancer Inst.

[20] Ascunce N, Salas D, Zubizarreta R, Almazán R, Ibáñez J, Ederra M (2010). Cancer screening in Spain. Ann Oncol Off J Eur Soc Med Oncol ESMO.

[21] Perry N, Broeders M, de Wolf C, Törnberg S, Holland R, von Karsa L (2008). European guidelines for quality assurance in breast cancer screening and diagnosis. Fourth edition--summary document. Ann Oncol Off J Eur Soc Med Oncol ESMO.

[22] Charlson ME, Pompei P, Ales KL, MacKenzie CR (1987). A new method of classifying prognostic comorbidity in longitudinal studies: development and validation. J Chronic Dis.

[23] Goldhirsch A, Wood WC, Coates AS, Gelber RD, Thürlimann B, Senn H-J (2011). Strategies for subtypes--dealing with the diversity of breast cancer: highlights of the St. Gallen International Expert Consensus on the Primary Therapy of Early Breast Cancer 2011. Ann Oncol Off J Eur Soc Med Oncol ESMO.

[24] Lyman GH, Giuliano AE, Somerfield MR, Benson AB, Bodurka DC, Burstein HJ (2005). American Society of Clinical Oncology guideline recommendations for sentinel lymph node biopsy in early-stage breast cancer. J Clin Oncol Off J Am Soc Clin Oncol.

[25] Classification of Chronic Pain, Second Edition (Revised) - IASP [Internet]. [cited 2016 Jun 22]. Available from: http://www.iasp-pain.org/PublicationsNews/Content.aspx?ItemNumber=1673

[26] Breivik H, Collett B, Ventafridda V, Cohen R, Gallacher D (2006). Survey of chronic pain in Europe: prevalence, impact on daily life, and treatment. Eur J Pain Lond Engl.

[27] Hickey OT, Nugent NF, Burke SM, Hafeez P, Mudrakouski AL, Shorten GD (2011). Persistent pain after mastectomy with reconstruction. J Clin Anesth.

